# Safety and efficacy of antiviral combination therapy in symptomatic patients of Covid-19 infection - a randomised controlled trial (SEV-COVID Trial): A structured summary of a study protocol for a randomized controlled trial

**DOI:** 10.1186/s13063-020-04774-5

**Published:** 2020-10-20

**Authors:** Prasan Kumar Panda, Arkapal Bandyopadhyay, Budha Charan Singh, Bikram Moirangthem, Gaurav Chikara, Sarama Saha, Yogesh Arvind Bahurupi

**Affiliations:** 1 General Medicine, AIIMS Rishikesh, Rishikesh, India; 2 Department of Pharmacology, AIIMS Rishikesh, Rishikesh, India; 3 Department of Medicine, AIIMS Rishikesh, Rishikesh, India; 4 Biochemistry, AIIMS Rishikesh, Rishikesh, India; 5 Community and Family Medicine, AIIMS Rishikesh, Rishikesh, India

**Keywords:** COVID-19, Hydroxychloroquine, Lopinavir/Ritonavir combination, Protocol, Randomised controlled trial, Ribavirin

## Abstract

**Objectives:**

1. To compare the safety and efficacy of Hydroxychloroquine with Ribavirin and standard treatment in patients with non-severe COVID-19 infection

2. To compare the safety and efficacy of standard treatment, Lopinavir-ritonavir with Ribavarin, and Hydroxychloroquine with Ribavirin in patients with severe COVID-19 infection

**Trial design:**

The study is an Open label, Parallel arm design, stratified randomised controlled trial. Patients will be categorised as non-severe or severe based on predefined criteria. Those who satisfy all inclusion criteria and no exclusion criteria in the respective categories, will be randomly assigned to one of the three treatment groups in a ratio of 1:1 in the non-severe category and 1:1:1 in the severe category.

**Participants:**

The trial will be undertaken in a tertiary care center of the country where both Covid and non-Covid patients are getting treated. All patients who are confirmed positive and admitted will be screened for the eligibility criteria and will be enrolled in the study after a written informed consent. Patients will be categorised as non-severe or severe based on predefined criteria.

**Inclusion Criteria (all required):**

1. Age ≥18 years at time of participation in the study

2. Laboratory (RT-PCR) confirmed infection with SARS-CoV-2

3. Symptomatic (severe or non-severe) Covid-19 disease

4. Willingness of study participant to accept randomization to any assigned treatment arm

**Exclusion Criteria:**

1. Use of medications that are contraindicated with Lopinavir/Ritonavir, Hydroxychloroquine/Chloroquine, or Ribavirin and that cannot be replaced or stopped

2. Patient already on antiretroviral therapy with Lopinavir-Ritonavir based regimen or on Hydroxychloroquine/Chloroquine or on Ribavirin

3. Any known contraindication to test drugs such as retinopathy and QT prolongation

4. Known allergic reaction or inability to take orally of Lopinavir-ritonavir, Hydroxychloroquine/ Chloroquine, Ribavarin

5. Pregnant or breastfeeding females

6. Receipt of any experimental treatment for 2019-nCoV (off-label, compassionate use, or trial related) within 30 days prior to participation in the present study or want to participate after enrolment

**Intervention and comparator:**

Two therapeutic interventions for non-severe category and three for severe category as described below

**Non-severe Treatment arms (NS-group):**

**Table:** 

**Treatment Arm**	**Drug**
A	Standard Treatment (ST_NS_)
B	Hydroxychloroquine 400 mg twice on first day followed by 400 mg per oral daily for 10 days + Ribavirin (1.2 g orally as a loading dose followed by 600mg orally every 12 hours) for 10 days + Standard Treatment (ST_NS_)

**Standard Treatment for non-severe cases (STNS):** Strict Isolation, Standard Precautions (Hand hygiene, Cough Etiquette, Wear surgical mask), Hydration, Proper Nutrition, Supportive Pharmacotherapy (Antipyretic, Antiallergic, Cough Suppressant), Treatment of Comorbid Diseases, Oseltamivir (75 mg BD) for patients who are tested positive for H1N1.

**Severe group Treatment arms (S-group):**

**Table:** 

**Treatment Arm**	**Drug**
A	Standard Treatment (ST_s_)
B	Hydroxychloroquine 400mg BD on day1 followed by 400 mg once daily + Ribavirin (1.2 g orally as a loading dose followed by 600mg orally every 12 hours) for 10 days + Standard Treatment (ST_s_)
C	Lopinavir(200mg) + Ritonavir (50mg) two tablets twice daily+ Ribavirin (1.2g orally as a loading dose followed by 600mg orally every 12 hours) for 10 days + Standard Treatment (ST_s_)^6^

**Standard Treatment for severe patients (STs):** Strict Isolation, Standard Precautions (Hand hygiene, Cough Etiquette, Wear surgical mask), Fluid Therapy, Supportive Pharmacotherapy (Antipyretic, Antiallergic, Cough Suppressant), Oxygen supplementation (As required), Invasive ventilation (As required), Antibiotic agents for other associated infections (according to 2019 ATS/IDSA guidelines for non-ICU and ICU patients), Vasopressor support, Renal-replacement therapy, Treatment of Comorbid Diseases, Oseltamivir (75 mg BD) for patients who are tested positive for H1N1.

**Main Outcomes:**

**Primary endpoints:** (1) Time to Clinical recovery (TTCR) defined as the time (in hours) from initiation of study treatment (active or placebo) until normalisation of fever, respiratory rate, oxygen saturation, and alleviation of cough, sustained for at least 72 hours.

(2) Time to SARS-CoV-2 RT-PCR negative in upper respiratory tract specimen, time to laboratory recovery of each organ involvement.

**Secondary Endpoints:** All causes mortality, Frequency of respiratory progression (defined as SPO2≤ 94% on room air or PaO2/FiO2 <300mmHg and requirement for supplemental oxygen or more advanced ventilator support), time to defervescence (in those with fever at enrolment), frequency of requirement for supplemental oxygen or non-invasive ventilation, frequency of requirement for mechanical ventilation, frequency of serious adverse events as per DAIDS table grade of severity.

Outcomes are monitored for 28 days from the time of enrolment into the study OR until the patient is discharged or death whichever is longer.

**Randomization:**

The randomization will be done using a secured central computer-based randomization using a secure website using a central, computer-based randomisation program in a ratio of 1:1 in the non-severe category and 1:1:1 in the severe category.

**Blinding (masking):**

This is an open labelled study i.e. Study assigned treatment will be known to the research team, the investigators and participants.

**Numbers to be randomised (sample size):**

Since it is an exploratory trial as COVID-19 being a new disease, all patients who came under the purview of the inclusion criteria within the study period (5 months duration of the recruitment period of the total 6 months duration of the study i.e. from the month of June, 2020 to October 2020) and who have consented for the study will be included.

**Trial Status:**

Protocol version:1.0

Recruitment start: June 3^rd^, 2020 (Ongoing)

Recruitment finish (expected): October 31^st^, 2020

**Trial registration:**

Clinical Trial Registry of India (CTRI): CTRI/2020/06/025575. Registration on 03 June 2020

**Full protocol:**

The full protocol is attached as an additional file, accessible from the Trials website (Additional file 1). In the interest in expediting dissemination of this material, the familiar formatting has been eliminated; this letter serves as a summary of the key elements of the full protocol.

## Supplementary information

**Additional file 1.** Full Study Protocol.

## Data Availability

Not applicable

