# Supplementary material for: Safety and efficacy of antiviral combination therapy in symptomatic patients of Covid-19 infection - a randomised controlled trial (SEV-COVID Trial): A structured summary of a study protocol for a randomized controlled trial
Source: Trials. 2020 Oct 20;21:866. doi: 10.1186/s13063-020-04774-5 (PMC7573526; doi:10.1186/s13063-020-04774-5)
Supplement: Supplementary file 1 — Additional file 1. Full Study Protocol. [file 13063_2020_4774_MOESM1_ESM.docx]

**S**afety and **e**fficacy of anti**v**iral therapy alone or in combination in symptomatic patients of **Covid**-19 infection - a randomised controlled trial (**SEV-Covid Trial**)

**Contents**

| **Topic** | **Page no.** |
| --- | --- |
| Research project submission form | 2-24 |
| Budgetary requirement | 25 |
| Undertaking by PI | 26 |
| Participant Information Sheet | 27-33 |
| Informed consent | 34-37 |
| Case report form | 38-51 |
| Adverse Event Log | 52 |

**Research Project Submission Form**

**A. Identification details:**

| **Code No.** (To be filled by the Research cell): |
| --- |
| **Type of project**: Intramural [�] Extramural [ ]  Drug trial/devise [ ] Collaborative [ ] Other [ ]  **Status of review**: New [�] Revised [ ]  **Proposal Title:** “Safety and efficacy of antiviral therapy alone or in combination in symptomatic patients of Covid-19 infection - a randomised controlled trial (SEV-Covid Trial)”  **Full Title of the Trial:** “Safety and efficacy of antiviral therapy alone or in combination in symptomatic patients of Covid-19 infection - a randomised controlled trial (SEV-Covid Trial)” |

**B. Investigator details:**

|  | **Name, Designation & Qualifications** | **Departmental Tel Nos. & Email ID** |
| --- | --- | --- |
| **Principal Investigator** | Dr. Prasan Kumar Panda  Assistant Professor, General Medicine, AIIMS Rishikesh | 9868999488  prasan.med@aiimsrishikesh.edu.in |
| **Co- investigator** | Dr. Girish Sindhwani  Professor and Head, Pulmonary Medicine, AIIMS Rishikesh | 7895050321  girish.sindhwani75@gmail.com |
| **Co- investigator** | Dr Shailendra Handu  Professor  Pharmacology, AIIMS Rishikesh | 8800847105  shailendra.handu@gmail.com |
| **Co- investigator** | Dr. Gaurav Chikara  Assistant Professor, Pharmacology, AIIMS Rishikesh | 9587262037  chikaraicon.gaurav@gmail.com |
| **Co- investigator** | Dr. Sarama Saha  Additional Professor, Biochemistry, AIIMS Rishikesh | 7055650111  sarama.bchem@aiimsrishikesh.edu.in |
| **Co- investigator** | Dr. Minakshi Dhar  Additional Professor,  General Medicine  AIIMS Rishikesh | 7060002778  minakshi.dhar@rediffmail.com |

| **Co- investigator** | Dr. Mukesh Bairwa  Assistant Professor,  General Medicine  AIIMS Rishikesh | 8130023989  drmukeshbairwa1982@gmail.com |
| --- | --- | --- |

| **Co- investigator** | Dr. Ankit Agarwal,  Associate Professor,  Anaesthesiology,  AIIMS Rishikesh | 8475000280  Ankit.anaes@aiimsrishikesh.edu.in |
| --- | --- | --- |

| **Co- investigator** | Dr. Yogesh Arvind Bahurupi  Assistant Professor, Community and Family Medicine, AIIMS Rishikesh | 9655007473 dr.yogeshab@gmail.com |
| --- | --- | --- |

| **Co- investigator** | Dr. Ramanuj Samanta  Assistant Professor,  Ophthalmology,  AIIMS Rishikesh | 8872292345  ramanuj.samanta@gmail.com |
| --- | --- | --- |
| **Co- investigator** | Dr. Rohit Walia  Assistant Professor,  Cardiology,  AIIMS Rishikesh | 8800492549  rohit.cardio@aiimsrishikesh.edu.in |
| **Co- investigator** | Dr. Itish Patnaik  Additional Professor,  Gastroenterology,  AIIMS Rishikesh | 8825564938  patnaik.itish@gmail.com |
| **Co- investigator** | Dr. Venkatesh Srinivasa Pai,  Additional Professor  Department of Rheumatology,  AIIMS Rishikesh | 9412312310  [drsvpai@yahoo.com](mailto:drsvpai@yahoo.com) |
| **Co- investigator** | Dr. Deepjyoti Kalita  Associate Professor,  Microbiology,  AIIMS Rishikesh | 7351962771  deep.micro@aiimsrishikesh.edu.in |

| **Co- investigator** | Dr. Puneet Gupta  Assistant Professor,  Microbiology,  AIIMS Rishikesh | 8894867079  puneet.micro@aiimsrishikesh.edu.in |
| --- | --- | --- |

| **Co- investigator** | Dr. Bharat Bhusan Bhardwaj  Assistant Professor,  Emergency Medicine,  AIIMS Rishikesh | 7800001352  bharat1352@yahoo.in |
| --- | --- | --- |

| **Co- investigator** | Mr Girraj Saini,  Nursing officer,  Division of Nursing,  AIIMS Rishikesh | 99820 70163  grajsaini.1991@yahoo.com |
| --- | --- | --- |

| **Co- investigator** | Dr. Ravi Gupta  Additional Professor,  Psychiatry,  AIIMS Rishikesh | 9458942135  [sleepdoc.ravi@gmail.com](mailto:sleepdoc.ravi@gmail.com) |
| --- | --- | --- |
| **Senior research fellow (SRF)** | Dr Arkapal Bandyopadhyay,  Clinical Pharmacology Resident, Department of Pharmacology,  AIIMS Rishikesh | 8820666435  drarkapal@gmail.com |
| **Junior research fellow (JRF-1)** | Dr Budha Charan Singh  Resident, Department of Medicine,  AIIMS Rishikesh | 8800103011  [budhaoinam@gmail.com](mailto:budhaoinam@gmail.com) |
| **Junior research fellow (JRF-2)** | Dr Bikram Moirangthem  Resident, Department of Medicine,  AIIMS Rishikesh | 8955838453  bkrajmoirangthem@gmail.com |

**C. Sponsor Information: Applicable [ ] Not applicable [**�**]**

| 1. **Name of sponsor/CRO:** |
| --- |
| 1. **Indian: a) Government** ⬜ Central ⬜ State ⬜ Institutional ⬜   **b) Private** ⬜ |
| 1. **International:** Government ⬜ Private ⬜ UN Agencies ⬜ |
| 1. **Industry:** National ⬜ Multinational ⬜ |
| 1. **Contact address of sponsor/CRO:** |
| 1. **Budget: Rs._____________________** 2. **Details of allocation of budget in Clinical Trial Agreement:** Yes [ ] No [ ] |

**D. Study related Information:**

| 1. **Design of study:** Epidemiological ⬜ Basic Sciences ⬜ Behavioral ⬜   Clinical ⬜ Interventional � ✔ Single Centre ⬜ Multicentre ⬜ |
| --- |
| 1. **No. of participants: AIIMS, Rishikesh Total (if multicentre)**   **Patient total = all patients who presented within the study period**  **Control**   1. **Duration of study: 6 months** 2. **Duration of subject participation (no. of visit by a participant in study): 28 days OR till patient is discharged or death whichever is longer ✔** |

**E. Interventional study: Yes [**�✔**] No [ ] If No, move to next section i.e. F**

| 1. **Does the study involve use of?**   Drugs ✔✔ Devices ⬜ Vaccines ⬜ Radiopharmaceutical ⬜  Recombinant DNA/Gene therapy ⬜ Stem cell ⬜ *(need BARC approval)*  *(need DBT-* *GEAC approval (NAC-SCRT registration and approval)*  Indian Systems of Medicines/ Alternate systems of Medicine ⬜  Any Other ⬜ None ⬜ |
| --- |
| 1. **Is it approved and marketed?**   In India �✔ UK & Europe � USA � Other Countries, Specify_______ |
| 1. **Does it involve a change in use, dosage, route of administration?** ✔ Yes ⬜ No   **If yes,** whether DCGI’s/Any other Regulatory Authority’s Permission is obtained?  Yes ⬜ No ⬜ ✔  **If yes,** copy of permission attached. Yes ⬜ No ⬜ |
| 1. **Is it an Investigational New Drug?** Yes ⬜ No � ✔   **If yes**   1. Investigator’s Brochure enclosed Yes ⬜ No ⬜ 2. Preclinical studies data available (If yes, provide summary) Yes ⬜ No ⬜ 3. Clinical studies data available (If yes, provide summary) Yes ⬜ No ⬜ 4. Clinical study is Phase I ⬜ Phase II ⬜ Phase III ⬜ Phase IV ⬜ NA ⬜   **If phase I-III** will the drug/device be provided free? Yes ⬜ No ⬜  **If phase IV** will the drug/device be provided at cost less than Yes ⬜ No ⬜  Hospital pharmacy?   1. DCGI’s permission obtained Yes ⬜ No ⬜   **If yes,** copy of letter enclosed Yes ⬜ No ⬜   1. Registered with Clinical Trial Registry – India Yes ⬜ No ⬜ 2. If yes copy of certificate enclosed Yes ⬜ No ⬜ |
| 1. **Data monitoring** 2. Is there a plan for reporting of adverse events? Yes ✔� No ⬜   **If yes**, reporting will be done to Sponsor ⬜ IEC � ✔   1. Is there a plan for interim analysis of data? Yes✔ � No ⬜ |
| 1. **Provision for travel/treatment due to injury out of study** Yes ⬜ No � ✔   **If yes**, by Sponsor ⬜ Investigator ⬜ Insurance Company ⬜ Any Other ⬜ |

**F. Details of participant of study:**

| **1. Will subjects from both sexes be recruited:** Yes [�✔] No [ ] |
| --- |
| 1. **Inclusion/exclusion criteria given:** Yes [�✔] No [ ] |
| 1. **Type of subjects:** Volunteers [] Patients [�✔ ] |
| 1. **Vulnerable subjects** Yes [ ] No [�✔]   (if **Yes** tick the appropriate boxes)  Pregnant Women [ ] Children [ ] Elderly [ ] Foetus [ ]  Illiterate [ ] Handicapped [ ] Terminally ill [ ] Seriously ill [ ]  Mentally challenged [ ] Economically & socially backward [ ] Any other [ ] |
| 1. **Special group subjects:** Yes [ ] No [�✔]   ( if **Yes** tick the appropriate boxes)  Captives [ ] Institutionalized [ ] Employees [ ] Students [ ]  Nurses/Dependent [ ] Staff [ ] Armed Forces [ ] Any Other [ ] |

G. **Privacy and confidentiality:**

| 1. **Study Involves** Direct Identifiers (Pt. identified by name/Cr. no.) ✔ �   Indirect Identifiers/Coded (Pt. identified after break of code) ⬜  Completely Anonymised /Delinked (Pt. cannot identified) ⬜   1. **Confidential handling of data by staff** Yes✔ � No ⬜ |
| --- |

**H. Detail of sample collection (If no sample being collected, move to next section i.e. I):**

| **A. *Regarding sample collection: NA***   1. **Collection of organs or body fluids or blood. If yes, please specify** Yes ✔ No ⬜   **Type: BLOOD**  **Amount each time: 06ml**  **No. of time: Every 72 hours**   1. **Collection of fetal tissue or abortus. If yes, please specify** Yes ⬜ No ⬜ 2. **Use of pre-existing/stored/left over samples. If yes, please specify** Yes ⬜ No ⬜   **____________________________________________________________________________________________________________________________**   1. **Proper disposal of material** Yes ⬜ No ⬜   ***B. Special situation***   1. **Will any sample collected from the patients be sent abroad?** Yes ⬜ No�   If yes, give details and address of collaborators  **______________________________________________________________**   1. ***Sample will be sent abroad because (Tick appropriate box)***   Facility not available in India ⬜  Facility in India inaccessible ⬜  Facility available but not being accessed ⬜  If so,reasons_________________________________________________________________   1. ***Has necessary clearance been obtained*** Yes ⬜ No ⬜ 2. **Collection for banking/future research** Yes ⬜ No ⬜ |
| --- |

1. **Participant Information Document (PID) and Consent Form:**

| 1. **Consent** *Written � ✔ Oral ⬜ Audio-Visual ⬜   Patient Information documents and consent form attached : (Tick the included elements)  Understandable language � Alternatives to participation �  Statement that study involves research � Confidentiality of records �  Sponsor of study � Contact information �  Purpose and procedures � Statement that consent is voluntary�  Risks & discomforts � Right to withdraw �  Benefits � Benefit if any on future �  Consent for future use of material biological NA  Free supply of drug till it is not marketed in country if necessary: NA  Compensation for study related injury: NO  Translation of Participant Information Document (PID) in local Language   1. **If healthy volunteers, PID for them NA** Yes No ⬜ 2. **If participant is child, PID for parent** : NA Yes ⬜ No ⬜ 3. **PID and Assent Form for child 8-18 yrs: NA** Yes ⬜ No ⬜ 4. **Consent form** in English � Local Languages �   (For participant/healthy volunteer/parent/legal guardian)   1. **Who will obtain consent?** PI-Co-PI � Nurse/Counselor ⬜   Research Staff ⬜ Any Other ⬜  ****If written consent is not obtained, give reasons……………………………………………...*** |
| --- |

| 1. **Will any advertising be done for recruitment of Subjects?** Yes ⬜ No �   (Posters, flyers, brochure, websites – if so attach a copy) |
| --- |

| 1. **For archival of record by Research Review cell for more** Yes ⬜ No � Not applicable ⬜   **than 5 years required**  If yes, for how many years……………………… |
| --- |

1. **Risk and benefits:**

| 1. **Is there physical/social/psychological risk/discomfort?** Yes ⬜ No �   **If yes**, Minimal or no risk ⬜  More than minimum risk ⬜  High risk ⬜   1. **Is there benefit** a) to the subject? Yes � No ⬜   Direct � Indirect ⬜  b) to the society Yes � No ⬜   1. **Do you think that the risk is in commensurate with the benefits to be accrued subjects/community/country?** Yes � No ⬜ 2. **Please identify the ethical issues involved in your study:** 3. High dose of chloroquine may have adverse effects; however, adverse effect monitoring will be done regularly. |
| --- |

| 1. **Do you have conflict of interest?** Yes ⬜ No �   **(Financial/Non financial)**  **If yes**, specify________________________________________________________________ |
| --- |

1. **Brief description of the proposal**

|  | Title of the project: **“Safety and efficacy of antiviral therapy alone or in combination in symptomatic patients of Covid-19 infection - a randomised controlled trial (SEV-Covid Trial)”** |
| --- | --- |
| (a) | Aim: To assess the safety and efficacy of Antiviral Therapy In Symptomatic Patients of COVID-19 Infection |
| (b) | Objectives:   1. To compare the safety and efficacy of Hydroxychloroquine with Ribavirin and standard treatment in patients of non-severe COVID-19 infection 2. To compare the safety and efficacy of standard treatment, Lopinavir-ritonavir with Ribavarin, and Hydroxychloroquine with Ribavirin in patients of severe COVID-19 infection |
| (c) | **Justification for study**: COVID 19 infection had become a pandemic. The presentation of the disease is highly variable. The treatment options for the disease are limited at present. For non-severe patient’s supportive treatment forms the mainstay of management. Various regimens have been tried in critically ill patients. Hydroxychloroquine, Lopinavir-ritonavir combination has been tried in clinical trials with limited success. The present study is planned to explore the combination of the above drugs with Ribavirin in different categories of COVID-19 infection. |
| (d) | **Scope of the Project**: The present study will evaluate the therapeutic potential of Hydroxychloroquine and Lopinavir-ritonavir in combination with Ribavirin in symptomatic cases of COVID-19 infection in a tertiary care hospital in India |
| (e) | Details of procedure and methodology proposed to be used (300 words) including description of the potential risks and benefits, exclusion and Inclusion criteria outcome measures, statistical analysis:  ***Patient population:*** *Covid-19 positive symptomatic cases either non-severe or severe category*  ***Intervention:*** *Two therapeutic interventions for non-severe category and three for severe category as described below*  ***Comparator:*** *Parallel arm design, compared among other interventional arm in same category*  ***Outcomes:*** *Clinical and laboratory improvements in 72 hours of treatment*  **Patient population-**  Patients diagnosed with SARS-CoV-2 infection after positive reverse-transcriptase–polymerase chain reaction (RT-PCR) assay.  **Inclusion criteria:**   1. Age ≥18 years at time of participation in the study 2. Laboratory (RT-PCR) confirmed infection with SARS-CoV-2 3. Symptomatic (severe or non-severe) Covid-19 disease 4. Willingness of study participant to accept randomization to any assigned treatment arm   **Exclusion criteria:**   1. Use of medications that are contraindicated with Lopinavir/Ritonavir, Hydroxychloroquine/Chloroquine, or Ribavirin and that cannot be replaced or stopped 2. Patient already on antiretroviral therapy with Lopinavir-Ritonavir based regimen or on Hydroxychloroquine/Chloroquine or on Ribavirin 3. Any known contraindication to test drugs such as retinopathy and QT prolongation 4. Known allergic reaction or inability to delivery oraly to Lopinavir-ritonavir, Hydroxychloroquine/ Chloroquine, Ribavarin 5. Pregnant or breastfeeding females 6. Receipt of any experimental treatment for 2019-nCoV (off-label, compassionate use, or trial related) within 30 days prior to participation in the present study or want to participate after enrolment   **Potential benefits:**   1. All those who participate in the trial will benefit from possible effective treatment for COVID-19 infection 2. The study will highlight the therapeutic options in management of the symptomatic patients of COVID-19 infection   **Potential risks:**  The present study will be conducted on symptomatic patients of COVID-19 attending AIIMS Rishikesh. The patients will be categorised based on severity and treatment will be allocated accordingly.  Adverse drug reactions, though not frequent may be associated with the treatment. Proper supportive management will be given to the patients at the institute.  **Methodology**:  **Flow Diagram**  Patients of COVID-19 infection - After confirmation by RT-PCR at AIIMS, Rishikesh  Screening for inclusion in trial Enrolment Excluded  ♦  Not meeting inclusion criteria  ♦  Declined to participate  ♦  Other reasons  Categorisation based on severity of patient  **SEVERE group**  **NON-SEVERE group**  Randomisation to treatment arm:  **A. Standard Treatment (ST-S)**  **B. Hydroxychloroquine + Ribavirin + ST-S**  **C. Lopinavir+ Ritonavir + Ribavirin + ST-S**  Randomisation to treatment arm:   1. **Standard Treatment (ST-NS)** 2. **Hydroxychloroquine + Ribavirin + ST-NS**  RandomisationAllocation Monitor progress of patient in 72 hours Follow-Up Patient improve- continue same treatment  Clinical evaluation- @72 hours  Improve - Continue same treatment  Progress – achieved end point and Shift to institute protocol for further treatment  Patient do not improve/ Signs of severe disease  Shift to **SEVERE group** treatment arms after randomization with new consent Analysis Analysis  **Total duration of observation**:  28 days from the time of enrolment into the study OR till patient is discharged or death whichever is longer  **Design:** Open label, Parallel arm design, stratified randomised controlled trial:  Patients will be screened for the study after diagnosis of COVID-19 infection by Microbiology team. Patients will be categorised as non-severe or severe based on predefined criteria. The patient will be enrolled in the study after a written informed consent. Since it is an exploratory trial as COVID-19 being a new disease, all patients who came under the purview of the inclusion criteria within the study period and who have consented for the study will be included. Clinical evaluations will be done by a separate investigator. Blinding will not be performed but dynamic randomization will be done. Patients will be followed up for a period of 28 days from the day of enrolment.  At the time of enrolment, demographic information will be collected from participants, and relevant data on their medical histories, comorbidities, and risk factors for severe COVID-19. It will be documented along with investigations including CBC, ECG, LFT, RFT, G6PD status.  The patients will be categorised as severe based on the following criteria:   - Confirmed pneumonia on chest imaging, SPo2 <93%, Pao_2_:Fio_2_ <300 mg Hg - Respiratory failure (need of mechanical ventilation) - Septic shock - Multiple (>2) organ dysfunction syndrome (MODS) - Liver disease (Child Pugh score ≥ C, AST>5 times upper limit) - Patients with known renal impairment (estimated glomerular filtration rate ≤30 mL/min/1.73 m2) or receiving continuous renal replacement therapy, haemodialysis, peritoneal dialysis - Other single organ failure (e.g. heart, pancreas, etc) if specific definition meets   Non-severe category includes mild and moderate disease severity category other than severe ones.  **Treatment Schedule**  **Non-severe Treatment arms (NS-group):**   \| **Treatment Arm** \| **Drug** \| \| --- \| --- \| \| A \| Standard Treatment (ST_NS_) \| \| B \| Hydroxychloroquine 400 mg twice on first day followed by 400 mg per oral daily for 10 days + Ribavirin (2.4 g orally as a loading dose followed by 1.2 g orally every 12 hours) for 10 days + Standard Treatment (ST_NS_) ^6^ \|   **Standard Treatment for non-severe cases (ST_NS_):**   1. Strict Isolation 2. Standard Precautions (Hand hygiene, Cough Etiquette, Wear surgical mask) 3. Hydration 4. Proper Nutrition 5. Supportive Pharmacotherapy (Antipyretic, Antiallergic, Cough Suppressant) 6. Treatment of Comorbid Diseases 7. Oseltamivir (75 mg BD) for patient who are tested positive for H1N1   If the patient improves clinically the same treatment will be continued. If the patient do-not improve or shows sign of severity (mentioned above) the patient will be shifted to the severity arm (S-group) of the clinical trial and again randomization will be done and enrolled after new consent only for severe group. However, if intervention falls on HCQ arm, then it will be taken as end point reached and shifted to institute treatment protocol for further management.  **Severe group Treatment arms (S-group):**   \| **Treatment Arm** \| **Drug** \| \| --- \| --- \| \| A \| Standard Treatment (ST_s_) \| \| B \| Hydroxychloroquine 400mg BD on day1 followed by 400 mg once daily + Ribavirin (2.4 g orally as a loading dose followed by 1.2 g orally every 12 hours) for 10 days + Standard Treatment (ST_s_) \| \| C \| Lopinavir(400mg) + Ritonavir (100mg) two tablets twice daily+ Ribavirin (2.4 g orally as a loading dose followed by 1.2 g orally every 12 hours) for 10 days + Standard Treatment (ST_s_)^6^ \|   **Standard Treatment for severe patients: (ST_s_)**   1. Strict Isolation 2. Standard Precautions (Hand hygiene, Cough Etiquette, Wear surgical mask) 3. Fluid Therapy 4. Supportive Pharmacotherapy (Antipyretic, Antiallergic, Cough Suppressant) 5. Oxygen supplementation (As required) 6. Invasive ventilation (As required) 7. Antibiotic agents for other associated infections (according to 2019 ATS/IDSA guidelines for non-ICU and ICU patients) 8. Vasopressor support 9. Renal-replacement therapy 10. Treatment of Comorbid Diseases 11. Oseltamivir (75 mg BD) for patient who are tested positive for H1N1   Patients will be assessed for clinical and laboratory improvements in 72 hours of treatment. In case the patient does not respond to the current treatment regimen the patient will be shifted to institute protocol for current practice of treatment.  **Timelines of investigations: Repeated at 72hrs interval**   \| Investigations \| Baseline \| Next 72hrs \| \| --- \| --- \| --- \| \| CBC \| **✔** \|  \| \| LFT \| **✔** \|  \| \| KFT \| **✔** \|  \| \| HBA1C and Blood sugar \| **✔** \|  \| \| RT-PCR for SARS-Cov-2 (until 24hrs apart twice negative) \| **✔** \|  \| \| PT/INR \| **✔** \|  \| \| Serum Electrolytes \| **✔** \|  \| \| ABG \| **✔** \|  \| \| Chest Xray \| **✔** \|  \| \| ECG \| **✔** \|  \| \| Other organ markers as per involvement \| **✔** \|  \|     **Dose Modifications:**  If any serious adverse event is recorded, the participant will be withdrawn from the trial and management will be done accordingly as per institute policy.  **Recording adverse events:**  All adverse events will be recorded in the medical records in the first instance. All adverse events will be recorded with clinical symptoms and accompanied with a simple, brief description of the event, including dates as appropriate. Each adverse event will be assessed for severity, causality, seriousness and expectedness and informed to IEC within 24hours.  **Discontinuation/withdrawal of participants:**  A participant may be withdrawn from trial treatment whenever continued participation is no longer in the participant’s best interests, but the reasons for doing so must be recorded.  Reasons for discontinuing treatment may include:   1. Unacceptable drug toxicity 2. Development of progressive disease 3. Patients withdrawing consent to further trial treatment   **Endpoints:**  **Primary endpoints:**   1. Time to Clinical recovery (TTCR)    - TTCR is defined as the time (in hours) from initiation of study treatment (active or placebo) until normalisation of fever, respiratory rate, and oxygen saturation, and alleviation of cough, sustained for at least 72 hours.    - Normalisation and alleviation criteria: 2. Fever - ≤36.9°C or -axilla, ≤37.2 °C oral 3. Respiratory rate - ≤24/minute on room air 4. Oxygen saturation - >94% on room air 5. Cough- mild or absent on a patient reported scale of severe, moderate, mild, absent 6. Time to SARS-CoV-2 RT-PCR negative in upper respiratory tract specimen 7. Time to laboratory recovery of each organ involvement   **Secondary Endpoints:**   1. All causes mortality 2. Frequency of respiratory progression  - Defined as SPO2≤ 94% on room air or PaO2/FiO2 <300mmHg and requirement for supplemental oxygen or more advanced ventilator support.  1. Time to defervescence (in those with fever at enrolment) 2. Frequency of requirement for supplemental oxygen or non-invasive ventilation 3. Frequency of requirement for mechanical ventilation 4. Frequency of serious adverse events as per AIDS table grade of severity   **Statistical analysis**:  All COVID-19 positive patients will be included in the study after informed consent. Since it is an exploratory trial as COVID-19 being a new disease, all patients who came under the purview of the inclusion criteria within the study period and who have consented for the study will be included. Data will be described as mean+ SD for most of the parameters. Comparisons of the descriptive variables of the two arms will be performed using the Wilcoxon rank sum test for continuous variables and the chi-square or Fisher’s exact test for categorical variables. Primary analysis will include all patients (Intention to treat) and sub-group populations (per-protocol).  **Ethical clearance:**  The present study deals with a new infectious disease which has no definite treatment. Current therapy is based on symptomatic treatment. The present study explores the possibilities of new drugs in SARS-CoV-2 infection. The study will be conducted after IEC approval. Written informed consent will be taken from all participants.  **Safety Outcomes**  Safety outcomes will be determined according to the division of AIDS table for grading severity of adult adverse events (<https://rsc.niaid.nih.gov/clinical-research-sites/daids-adverse-event-grading-tables>), with specific focus on the events of gastrointestinal tract, arrhythmias, myopathies. In case of serious ADRs including death, compensation will be given as per institute protocol. |
| *(f)* | *Detailed time line for the project:(details of duration required for procurement, data collection, analysis, preparation of final report,etc)*  Duration of procurement: None, already available  Data collection: 5 months  Analysis: 15 days  Preparation of final report: 15 days |
| (g) | Resume of relevant literature on the subject, with special reference to the areas in which information is lacking:  COVID-19 is an infectious disease caused by novel corona virus SARS-CoV-2. WHO has declared it as a pandemic.^[[1]](#footnote-1)^ The presentation of the disease ranges from asymptomatic to mild cough, fever and sore throat. Disease can occasionally progress to pneumonia with acute respiratory distress syndrome, sepsis and septic shock. Definitive treatment is not available for COVID-19 infection. Strict isolation, standard precautions like hand hygiene and cough etiquette along with supportive therapy forms the mainstay of management. Antibiotics has been used as a part of standard treatment. Supportive treatment forms the mainstay of non-severe cases of COVID-19 infections. Hydroxychloroquine (HCQ) is thought to be effective in treatment and prophylaxis.^2^ Global consensuses on its use has been discussed widely.^3^ Lopinavir-ritonavir combination has also been tried in hospitalised patients.^4,5^ Combination of both the regimens with Ribavirin seems to be a potential therapeutic option in symptomatic COVID infections.^6,7^ Several combination therapies have also been suggested in the newly published literature. No clear consensus or definitive treatment is present till date. The present study explores therapeutic options in symptomatic patients with COVID-19 infection. |
| (h) | Relevant References:   1. Corona Virus situation report. Available at- <https://www.who.int/emergencies/diseases/novel-coronavirus-2019/situation-reports>. 2. Advisory for prophylaxis of SARS-CoV-2 infections. Available at <https://www.mohfw.gov.in/pdf/AdvisoryontheuseofHydroxychloroquinasprophylaxisforSARSCoV2infection.pdf>. 3. Zhou D, Dai SM, Tong Q. COVID-19: a recommendation to examine the effect of hydroxychloroquine in preventing infection and progression [published online ahead of print, 2020 Mar 20]. *J Antimicrob Chemother*. 2020. 4. Bhatnagar T, Murhekar M. Lopinavir/ritonavir combination therapy amongst symptomatic coronavirus disease 2019 patients in India: Protocol for restricted public health emergency use. 5. Multicenter collaboration group of Department of Science and Technology of Guangdong Province and Health Commission of Guangdong Province for chloroquine in the treatment of novel coronavirus pneumonia. Expert consensus on chloroquine phosphate for the treatment of novel coronavirus pneumonia]. Zhonghua Jie He He Hu Xi Za Zhi. 2020 Mar 12;43(3):185-188. doi: 10.3760/cma.j.issn.1001-0939.2020.03.009 6. Landscape analysis of therapeutics as 21st March 2020. Available at: <https://www.who.int/blueprint/priority-diseases/key-action/Table_of_therapeutics_Appendix_17022020.pdf?ua=1>. Last accessed on 30^th^ March 2020. 7. Chen Z, Hu J, Zhang Z, Jiang S, Han S, Yan D, et al. Efficacy of hydroxychloroquine in patients with COVID-19: results of a randomized clinical trial. medRxiv. 2020 Mar 31;2020.03.22.20040758. |

**BUDGETARY REQUIREMENT:**

| Details of items with quantity | Study period (6 Months) | Total (Rs) |
| --- | --- | --- |
| Manpower | 1 SRF (Senior Resident, Department of Pharmacology)  1 JRF (Junior Resident, Department of General Medicine) | Nil since he/she is part of institute resident-ship |
| Study drugs | Available in the institute |  |
| Equipment | Available in the institute |  |
| Investigations: n= any till end of the study  ECG (Baseline, every 72 hours)  LFT (Baseline and every 72 hours)  KFT (Baseline and every 72 hours)  CBC (Baseline and every 72 hours)  Others specific marker of an organ involvement | Available in the institute |  |
| Chemicals/ Reagents/ Consumables | Available in the institute |  |
| Contingencies | NA |  |
| Total |  |  |

Note: -

1. All expenditure should be logically justified by the proposed project
2. All purchases will be made as per GFR and institutional financial guidelines for research projects.

**Signature of PI**

**Name__________________________ Date_________________**

**All India Institute of Medical Sciences, Rishikesh (Uttarakhand)**
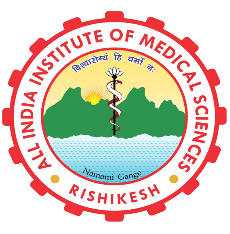


**Institutional Ethics Committee**

**संस्थागतनीतिसमिति**

**Undertaking**

This is to state that:

In submitted trial titled **“Safety and efficacy of antiviral therapy alone or in combination in symptomatic patients of Covid-19 infection - a randomised controlled trial (SEV-Covid Trial)”**

1. No expenses related to research work will be made by patient.

2. No similar Research work is being carried at AIIMS Rishikesh at this point of time.

3. Research trial with similar Title and objectives has not been submitted to IEC before/has not been approved by IEC, AIIMS Rishikesh previously.

4. Principal Investigator will maintain record of UHID of all Patients/Subjects (name, address and contact numbers in case of a survey/Questionnaire) participating in the study and submit record of the same in Institutional Ethics Committee, after completion of the study.

**Signature & Name of PI: PRASAN KUMAR PANDA**

**Date: 10/04/2020**

**Participant Information Sheet (PIS)**

| Title of Study: | **Safety and efficacy of antiviral therapy alone or in combination in symptomatic patients of Covid-19 infection - a randomised controlled trial (SEV-Covid Trial)** | | |
| --- | --- | --- | --- |
| Hospital or Institution: | **All India Institute of Medical sciences and research, Rishikesh** | Subject Name:  Contact No.: |  |
| Subject Initials: |  | Age/ Year of Birth:  Gender: |  |
|  |  |  |  |
| Height/Weight |  | BMI |  |
| Comorbidities at the time of enrolment |  | UHID |  |

We are going to brief you about the study to let you decide for participation.

You are being invited to participate in a research study. This form gives you important information about this study to help you decide if you want to take part in it.

Please take the time to read this information carefully. The study doctor will explain the details of this study to you and you can ask questions. You can also discuss this study with other people such as your family, any other doctor or another doctor if you yourself are one.

You can choose whether to take part in this study. You are free to say yes or no. If you do not want to participate, your regular medical care will not be affected. Taking part in this study will also not prevent a doctor from providing any medical care to you, if required. Even if you join this study, you may change your mind and stop participating at any time, or change in to prophylaxis arm of study without giving reason at any time.

If you are taking part in any other research study, you cannot join this study. If you decide to take part in this study, you will be asked to sign and date this form. You will receive a copy of the signed form.

**Rationale and purpose of study**

Over the past few months, a health crisis brought by the novel severe acute respiratory syndrome coronavirus-2 (SARS-CoV-2) has swept the world, taking a massive toll on people and nations. The virus rapidly causes mild to severe respiratory symptoms, and it is readily transmitted from human to human by droplet spread. The resulting infectious disease has been termed COVID-19. Most infections are mild and similar in severity to the common cold. However, about 15-20% of those with documented infection have more severe symptoms, and approximately 5% of those infected have life-threatening complications, including a severe acute respiratory syndrome, pneumonia, respiratory failure, sepsis, delirium, cardiogenic shock, acute kidney injury, liver injury, and multi-organ failure.

There is currently no vaccine for the novel coronavirus, nor has any pharmacological therapy been proven as effective for prevention, mitigation, or treatment. Combination therapy with Hydroxychloroquine, Lopinavir-ritonavir has been used for treatment

The current study is being conducted in AIIMS Rishikesh to investigate and get definitive answer on therapy of symptomatic COVID-19 infections.

**1. WHAT YOU SHOULD KNOW ABOUT THIS STUDY**

**a. Why is this study being done?**

This study is being done to evaluate the safety and efficacy of different doses of hydroxy-chloroquine, Lopinavir-ritonavir in combination therapy in treatment of COVID-19 infection

**b. How many people will take part in this study?**

The study plans to enrol approximately 165 patients at AIIMS Rishikesh.

**c. How long will you be participating in this study?**

Your participation in this study will last for 28 days from day of enrolment or till your discharge from hospital

**d. What study drug is being tested?**

The treatment schedules are as follows for each of the four groups:

Non-severe case- A. Standard treatment or B. Hydroxychloroquine + Ribavirin

Severe cases- A. Standard treatment, B. Hydroxychloroquine + Ribavirin,

C. Lopinavir-ritonavir + Ribavirin

**e. What are the chances you will receive the study drug?**

Participation in this study is voluntary. Those who consent for the study will be randomized in the various groups of intervention arms. To which group of intervention, you will go will be determined by chance alone, a process called randomization. Based on severity you will be allotted drugs as per randomisation schedule.

**f. Will you be enrolled in the study if you meet the eligibility criteria?**

You must satisfy the eligibility criteria for the study in order to be enrolled. For this you need to consent for the participation. After that, you will be examined against the eligibility criteria described in the study protocol. You will be enrolled in the study only if you satisfy the eligibility criteria.

**g. Will you know the study drug you receive if you meet the study requirements?**

No. The study doctor and the study staff will be fully aware of the study drug you are receiving.

**h. What will be asked of you if you take part in this study?**

If you agree to participate in the study, you will need to provide information necessary for assessing your eligibility in the study. You will need to be compliant with the study intervention. Assessments required to ascertain safety of the drug will be carried out from time to time. You will be investigated from time to time during the course of our admission. You will undergo laboratory testing including hemograms, LFT, KFT which will involve giving blood samples. You will be treated according to the severity of your disease

Besides these, you may experience an unexpected serious adverse event including death which may or may not be related to clinical trial intervention as may happen in any study.

**i. Are there any risks from taking part in this study?**

Hydroxychloroquine has been used in humans for management and prevention of malaria. The known side effects of the drug include headache, loss of appetite, nausea, vomiting, abdominal cramps, blurred vision or diarrhoea, some disturbance in ECG. While most of these are transient and reversible, vision disturbance may persist for long or may be irreversible. However, the doses at which the drug is being given in the study, the chances of vision disturbances are less than 1%. If you are pregnant or are breast feeding you should not take part in the study. Lopinavir Ritonavir can cause diarrhoea and abdominal cramps in some cases. The nature of side effect is usually mild. Ribavirin can be associated with bone marrow depression which may rarely lead to anaemia. We will be monitoring you during the study and any ADR will be managed appropriately

**j. Are there any benefits of taking part in this study?**

You may or may not get protection as health care team worker taking care of infected patents but are not expected to gain anything from this research. This research may be of use to society, especially healthcare professionals taking care of COVID-19 patients in future in decisive manner.

**2. FURTHER INFORMATION ON YOUR RIGHTS ON STUDY TERMINATION AND MANAGEMENT OF YOUR SAMPLES**

**a. Can you stop taking part in the study?**

Your participation is voluntary and you can withdraw from the study at any time without giving any reason. Your refusal to participate will not involve any penalty or loss of benefits to which you are otherwise entitled. Your study doctor may terminate your participation in the study if it is in your interest.

**b. How will your blood samples be stored and managed by AIIMS Rishikesh?**

You will have to undergo blood tests, ECG and other biochemical tests if required and AIIMS Rishikesh will use your samples for tests outlined in this form or for tests needed to ensure your safety. All your data will be kept confidential but can be disclosed to government agencies taking care of such projects or to the court of law. Only the investigators conducting this study will have access to your medical records.

**3. DETAIL SAFETY INFORMATION**

**SAFETY - POTENTIAL RISKS AND DISCOMFORTS**

**a. What are the likely risks with hydroxychloroquine, Lopinavir Ritonavir and Ribavirin?**

The medicine being administered to you is safe and has been used for managing and preventing malaria. However, as discussed previously, it may cause the following headache, loss of appetite, nausea, vomiting, abdominal cramps, blurred vision or diarrhoea, some disturbance in ECG. While most of these are transient and reversible, vision disturbance may persist for long or may be irreversible. However, the doses at which the drug is being given in the study, the chances of vision disturbances are less than 1%. Ritonavir-lopinavir is a commonly used antiretroviral drug which may cause gastrointestinal disturbance in some patients. Ribavirin is used in chronic hepatitis may cause anaemia.

**b. What are the risks of using hydroxychloroquine in combination with other drugs?**

Tell the study doctor or the study staff about any drugs you are taking, have recently taken or are planning to take, including herbal remedies, supplements, experimental therapies and drugs you take without a prescription. You will be expected to provide medical/personal history to the medical doctor correctly. Please discuss any concerns you may have with the study doctor.

**c. What are the risks associated with procedure done in this study?**

The known risks and side effects of study related tests or procedures are given here.

Blood Draws

You will have your blood drawn during the study. Possible side effects of having blood drawn are tenderness, pain, bruising, bleeding, and/or infection where the needle goes into the skin and blood vein. Having your blood drawn may rarely also cause to feel nauseated and/or lightheaded.

**d). Pre-treatment investigations**

For this study ECG, complete blood count, Liver function test, Renal function test, G6PD deficiency.

**4. POTENTIAL COSTS/REIMBURSEMENTS**

**a). What will this study cost you?**

Your participation in the study will not result in any cost to you. You will receive study medicine free of charge and there will be no charges for the visits, blood tests, or procedures required by the study.

**5. CONFIDENTIALITY**

**a). How will your medical records and information be kept confidential?**

Your medical records will be kept confidential as allowed by the applicable laws. If results of the study are published, your identity will not appear. Neither your results nor samples will be identified with your name. Information about your participation in this study will be associated with you by using subject identification number. The study doctor or his/her study staff with label off your samples with the identification number. Only the study doctor and his/her study staff will be able to link that number to you at all times.

**b). Who will have access to your medical information?**

If you take part in this study, information about your health will be collected and analysed by the study team. These people will have access to your medical information and see your name, other personal information such as year of birth and gender, but will be obliged to keep this information confidential unless required by law or a regulatory authority.

You have a right access to your information and the right to correct information, but cannot be given information about results from study procedures while the study is being conducted.

**6. COMPENSATION FOR INJURY**

**a). What do you do if you think you have an injury/illness related to your participation in this study?**

If you think you have an injury/illness that is related to the study, you should immediately notify **the Principal Investigator or co-investigator**, at AIIMS Rishikesh or one of the staff members working on the study. The investigator and the study staff may be reached. Contact number provided at the end of the document**.** The investigator and the study staff will make sure that you receive necessary treatment.

**b). If you have an injury/illness related to your participation in the study, will you be compensated in any way?**

In the event of an injury occurring to you during the clinical trial, you shall be provided free medical management as long as required or till such time it is established that the injury is not related to the trial, whichever is earlier. In the event of a trial related injury or death compensation shall be provided as per AIIMS Rishikesh policy.

**Informed Consent form**

By signing your name below, you confirm the following:

• You have read this form (or have had it read to you) and the study has been explained to you.

• You have been given a chance to ask any questions about the study, and your questions have been answered.

• You may ask questions at any time about this study

• You will be informed in a timely manner, of any information that may impact your willingness to continue participation in the study

• Your participation in the study is completely voluntary and you may leave the study at any time

• You have had all alternatives discussed with you.

• You do not give up any of your legal rights by signing this form

• You authorise access to your confidential information

• You authorise the processing of your information and samples

• You agree to participate in this study

• You will receive a copy of this form

**Agreement to participate:**

| Title of Study: | **Safety and efficacy of antiviral therapy alone or in combination in symptomatic patients of Covid-19 infection - a randomised controlled trial (SEV-Covid Trial)** | | |
| --- | --- | --- | --- |
| Hospital or Institution: | **All India Institute of Medical sciences and research, Rishikesh** | Subject Name: |  |
| Subject Initials: |  | Subject Year of Birth: |  |
|  |  |  | YYYY |

**Address of the Subject:**

**Occupation:** Student / Self-Employed / Service / Housewife /Others (Please tick as appropriate)

**Annual Income of the subject**:

**Name and address of the nominee(s) and his relation to the subject** (for the purpose of compensation in case of trial related death).

Name: Relation to subject:

Address:

| **Your consent** | **Subject Initials** |
| --- | --- |
| **1.** I confirm I have read (or have had it read to me) and understood the information sheet for the above Study and have had the opportunity to ask questions. I am above 18 years of age. |  |
| **2.** I understand that my participation is voluntary and that I am free to withdraw at any time, without giving any reason, without my medical care or legal rights being affected. |  |
| **3.** I understand that AIIMS Rishikesh, the Ethics Committee and Regulatory Authorities will not need my permission to look at my health records both in respect of the current Study and any further research that may be conducted in relation to it, even if I withdraw. I agree to this access. However, I understand that my identity will not be revealed in any information released to unauthorized third parties or published. I understand that two categories of disease are treated with five arms. I understand that one treatment I will receive as per randomisation. |  |
| **4.** I consent to the collection, processing, reporting of my personal and sensitive data for scientific healthcare and/or medical research and medical product development purposes. |  |
| **5.** I agree not to restrict the use of any data or results, which arise from this study, provided such use is only for scientific purpose (s). |  |
| **6.** I agree for the audio-visual recording of the conversation |  |
| **7.** I agree to take part in the above Study. |  |

DATE

Thumb impression/subject’s signature

Print subject’s name

DATE

Signature of the Investigator/person who conducted the informed consent discussion

PRINT name of the Investigator/person who conducted the informed consent discussion

Signature of Impartial Witness*

DATE

Print name of Impartial Witness*

*Address and contact:*

**Impartial witness is required if the subject is unable to read the consent.*

(Copy of the patient Information Sheet and duly filled Informed Consent Form shall be handed over to the subject or his/her attendant).

**Contact Information:**

**Principal Investigator (study doctor): Dr. Prasan Kumar Panda**

Dept. of Medicine AIIMS Rishikesh, 249203, Rishikesh, Uttarakhand, India

Contact Number: +91-9868999488

Email id: prasan.med@aiimsrishikesh.edu.in

**Co-Investigator: Dr. Gaurav Chikara**

Dept. of Pharmacology, AIIMS Rishikesh, 249203, Rishikesh, India

Contact Number: 9587262037

Email id: chikaraicon.gaurav@gmail.com

**Co-Investigator: Dr ANKIT AGARWAL**

Dept. of CCU, AIIMS Rishikesh, 249203, Rishikesh, India

Contact Number: 8475000292

Email id: puneet.phar@aiimsrishikesh.edu.in

**CASE REPORT FORM**

**PARTICIPANT ID I___I I___I I___I I___I I___I -- I___I I___I I___I I___I Date of enrolment [__][__]/[__][__]/[__][__][__][__]**

**Country ____________State___________________District_____________________Lane_________________**

**CLINICAL INCLUSION CRITERIA**

| One or more of these during this illness | A history of self-reported feverishness or documented fever | ☐Yes ☐No |
| --- | --- | --- |
|  | Cough | ☐Yes ☐No |
|  | Dyspnoea (shortness of breath) OR Tachypnoea | ☐Yes ☐No |
|  | Clinical suspicion of ARI despite not meeting criteria above | ☐Yes ☐No |

**DEMOGRAPHICS**

| Sex at Birth ☐Male ☐Female ☐Not specified Date of birth: [__][__]/[__][__]/[__][__][__][__]  If date of birth is unknown, record: Age [___][___][___]years  Healthcare Worker? ☐Yes ☐No ☐Unknown  Laboratory Worker? ☐Yes ☐No ☐Unknown  Pregnant? ☐Yes ☐No ☐Unknown ☐N/A If yes: Gestational weeks assessment [___][___] weeks |
| --- |

**CO-MORBIDITIES (existing prior to admission) (Unk = Unknown)**

| Chronic cardiac disease (not hypertension)  Yes ☐No ☐Unk | Diabetes ☐Yes ☐No ☐Unk |
| --- | --- |
| Hypertension Yes ☐No ☐Unk | Current smoking ☐Yes ☐No ☐Unk |
| Chronic pulmonary disease ☐Yes ☐No ☐Unk | Tuberculosis ☐Yes ☐No ☐Unk |
| Asthma Yes ☐No ☐Unk | Asplenia Y☐es ☐No ☐Unk |
| Chronic kidney disease ☐Yes ☐No ☐Unk | Malignant neoplasm ☐Yes ☐No ☐Unk |
| Chronic liver disease ☐Yes ☐No ☐Unk | Other ☐Yes ☐No ☐Unk  If yes, specify: ------------------------------------------------------------------------------------------------------ |
| HIV ☐Yes-on ART ☐Yes-not on ART ☐No ☐Unknown |  |

**MEDICATION Is the patient CURRENTLY receiving any of the following?**

| 1. Oral/orogastric fluids? ☐Yes ☐No ☐Unknown Intravenous fluids? ☐Yes ☐No Unknown 2. Antiviral? ☐Yes ☐No Unknown If yes: ☐Ribavirin ☐Lopinavir/Ritonavir ☐Neuraminidase inhibitor ☐Interferon alpha Interferon beta Other, specify: _________________________ 3. Corticosteroid? ☐Yes ☐No ☐Unknown If yes, route: ☐Oral ☐Intravenous Inhaled If yes, provide agent and maximum daily dose:____________ 4. Antibiotic? ☐Yes ☐No Unknown If yes, specify: ____________________ 5. Antifungal agent? ☐Yes ☐No Unknown, If yes, specify: ____________________ 6. Antimalarial agent? ☐Yes ☐No ☐Unknown If yes, specify____________________ 7. Experimental agent? ☐Yes ☐No ☐Unknown If yes, specify____________________ 8. Non-steroidal anti-inflammatory (NSAID) ☐Yes ☐No ☐Unknown 9. Angiotensin converting enzyme inhibitors (ACE inhibitors) ☐Yes ☐No ☐Unknown Angiotensin II receptor blockers (ARBs) ☐Yes ☐No ☐Unknown |
| --- |

**SUPPORTIVE CARE**

**Is the patient CURRENTLY receiving any of the following?**

| 1. ICU or High Dependency Unit admission? ☐Yes ☐No ☐Unknown 2. Oxygen therapy? ☐Yes ☐No ☐Unknown   If yes, complete all below O2 flow: ☐1-5 L/min ☐6-10 L/min ☐11-15 L/min ☐>15 L/min ☐Unknown  Source of oxygen: ☐Piped ☐Cylinder ☐Concentrator ☐Unknown  Interface: ☐Nasal prongs ☐HF nasal cannula ☐Mask ☐Mask with reservoir ☐CPAP/NIV mask Unknown   1. Non-invasive ventilation? (e.g.BIPAP/CPAP) ☐Yes ☐No ☐N/A 2. Invasive ventilation (Any)? ☐Yes ☐No ☐Unknown 3. Inotropes/vasopressors? ☐Yes ☐No ☐Unknown 4. Extracorporeal (ECMO) support? ☐ 5. Prone position? ☐Yes ☐No ☐Unknown |
| --- |

**Case Summary:**

**Travel and Contact history (if significant)**

**Physical examination: (with consent and after adequate precautions)**

1. **Mental status –**
2. **General appearance –**
3. **Vitals: PR - , RR - , BP - , Temp - , SpO_2_ -**
4. **Skin –**

| SYSTEM | Inspections | Palpations | Percussions | Auscultations |
| --- | --- | --- | --- | --- |
| 1. Chest (respiratory system) |  |  |  |  |
| 1. Chest (cardiovascular) |  |  |  |  |
| 1. Abdomen |  |  |  |  |

1. **Genito-urinary-Rectal exam –**
2. **Musculoskeletal Assessment –**
3. **Neurological Assessment –**

**LABORATORY RESULTS ON ADMISSION (*record units if different from those listed)**

| Parameter | Value | Not done | Parameter | Value | Not done |
| --- | --- | --- | --- | --- | --- |
| Haemoglobin (g/L) |  |  | Urine R/M (significant if any) |  |  |
| WBC count (x109/L |  |  | Sodium (mEq/L |  |  |
| Haematocrit (%) |  |  | Potassium (mEq/L) |  |  |
| Platelets (x109/L) |  |  | Calcium(mg/dL) |  |  |
| APTT/PT/INR |  |  | Lactate (mg/dL) |  |  |
| Total bilirubin (mg/dL) |  |  | Procalcitonin (ng/mL) |  |  |
| Direct bilirubin (mg/dL) |  |  | CRP (mg/L) |  |  |
| Albumin (g/dl) |  |  | LDH (U/L) |  |  |
| Globulin (g/dl) |  |  | Creatine kinase (U/L) |  |  |
| SGOT/SGPT (U/L) |  |  | Troponin (ng/mL) |  |  |
| Urea (mg/dL) |  |  | ESR (mm/hr) |  |  |
| Creatinine (mg/dL) |  |  | D-dimer (mg/L) |  |  |
| RBS |  |  | HbA1C |  |  |
| Others |  |  |  |  |  |

**Non-severe Treatment arms (NS-group)**

**PARTICIPANT ID I___I I___I I___I I___I I___I -- I___I I___I I___I I___I Date of enrolment [__][__]/[__][__]/[__][__][__][__]**

**Day of Hospitalization: _______________ Date of follow-up: [__][__]/[__][__]/[__][__][__][__]**

**VITAL SIGNS:**

| Temperature: °C Heart rate: beats per min Respiratory rate: breaths/min  BP: mmHg Hydration Status: Sternal capillary refill time >2seconds ☐Yes ☐No GCS:  Oxygen saturation (SPO2): % on ☐ room air ☐oxygen therapy ☐Unknown |
| --- |

Standard Treatment for non-severe cases (ST_NS_):

1. Strict Isolation: ☐Yes ☐No ☐Unk
2. Standard Precautions (Hand hygiene, Cough Etiquette, Wear surgical mask): ☐Yes ☐No ☐Unk
3. Nutrition: ☐Enteral ☐Parenteral ☐Unk
4. Supportive Pharmacotherapy (Antipyretic, Antiallergic, Cough Suppressant if any with days and frequency)

- ______________________________________________
- _______________________________________________
- ______________________________________________

1. Treatment of Comorbid Diseases(to mention condition and treatment given)
2. Oseltamivir (for patient who are tested positive for H1N1)

- Total number of days received:
- Dosage and Frequency:

1. Treatment Group:

☐Treatment Arm: A (Standard Treatment-ST_NS_)

☐Treatment Arm: B (Hydroxychloroquine 400 mg twice on first day followed by 400 mg per oral daily for 10 days + Standard Treatment -ST_NS_) ^^[[2]](#endnote-1)^^

**CLINICAL FEATURES (Unk = Unknown)**

| - Cough and sputum production - Sore throat - Chest pain - Shortness of breath - Confusion | ☐Yes ☐No ☐Unk  ☐Yes ☐No ☐Unk  ☐Yes ☐No ☐Unk  ☐Yes ☐No ☐Unk  ☐Yes ☐No ☐Unk | - Seizures - Vomiting - Nausea - Diarrhoea - Conjunctivitis - Myalgia - Other, specify_______ | ☐Yes ☐No ☐Unk  ☐Yes ☐No ☐Unk  ☐Yes ☐No ☐Unk  ☐Yes ☐No ☐Unk  ☐Yes ☐No ☐Unk  ☐Yes ☐No ☐Unk  ☐Yes ☐No ☐Unk |
| --- | --- | --- | --- |

**LABORATORY RESULTS ON FOLLOW UP (*record units if different from those listed)**

| Parameter | Value | Not done | Parameter | Value | Not done |
| --- | --- | --- | --- | --- | --- |
| Haemoglobin (g/L) |  |  | Urine R/M (significant if any) |  |  |
| WBC count (x109/L |  |  | Sodium (mEq/L |  |  |
| Haematocrit (%) |  |  | Potassium (mEq/L) |  |  |
| Platelets (x109/L) |  |  | Calcium(mg/dL) |  |  |
| APTT/PT/INR |  |  | Lactate (mg/dL) |  |  |
| Total bilirubin (mg/dL) |  |  | Procalcitonin (ng/mL) |  |  |
| Direct bilirubin (mg/dL) |  |  | CRP (mg/L) |  |  |
| Albumin (g/dl) |  |  | LDH (U/L) |  |  |
| Globulin (g/dl) |  |  | Creatine kinase (U/L) |  |  |
| SGOT/SGPT (U/L) |  |  | Troponin (ng/mL) |  |  |
| Urea (mg/dL) |  |  | ESR (mm/hr) |  |  |
| Creatinine (mg/dL) |  |  | D-dimer (mg/L) |  |  |
| Others |  |  |  |  |  |

Influenza virus: ☐Positive ☐Negative ☐Not done If positive, type ______________

Coronavirus: ☐ Positive ☐Negative ☐ Not done If positive: ☐ MERS-CoV ☐SARS-CoV-2 ☐Other

Other respiratory pathogen: ☐Positive ☐ Negative ☐Not done If positive, specify:___________

Other pathogen of public health interest detected: If yes, specify: __________________________

Radiological Investigations (if performed):

1. Chest X Ray/CT Performed (report):
2. Ultrasonography:
3. Others

Previous Treatment Regime (If any-to specify group with duaration):

☐Non-Severe Treatment arms (NS-group): Treatment Arm-A: ___________________________

Outcomes:

1. Clinical recovery ☐Yes ☐No
2. Time to 2019-nCoV RT-PCR negative in upper respiratory tract specimen in days (if applicable):
3. Time to defervescence in hours (if applicable)
4. Serious adverse events (specify if any)
5. **Final Clinician’s Impression:**

1. [↑](#footnote-ref-1)
2. **Severe Treatment arms (S-group)**

   **PARTICIPANT ID** I___I I___I I___I I___I I___I -- I___I I___I I___I I___I **Date of enrolment** [__][__]/[__][__]/[__][__][__][__]

   **Day of Hospitalization: _______________ Date of follow-up:** [__][__]/[__][__]/[__][__][__][__]

   **VITAL SIGNS:**

   | Temperature: °C Heart rate: beats per min Respiratory rate: breaths/min  BP: mmHg Hydration Status: Sternal capillary refill time >2seconds ☐Yes ☐No GCS: Oxygen saturation (SPO2): % on ☐room air ☐oxygen therapy ☐Unknown |
   | --- |

   **Standard Treatment for Severe cases (ST_NS_):**

   Strict Isolation: ☐Yes ☐No ☐Unk

   Standard Precautions (Hand hygiene, Cough Etiquette, Wear surgical mask): ☐Yes ☐No ☐Unk

   Nutrition: ☐Enteral ☐Parenteral ☐Unk

   Supportive Pharmacotherapy (Antipyretic, Antiallergic, Cough Suppressant if any with days and frequency)

   ______________________________________________

   _______________________________________________

   ______________________________________________

   Treatment of Comorbid Diseases(to mention condition and treatment given)

   Antibiotic agents for other associated infections (according to 2019 ATS/IDSA guidelines for non-ICU and ICU patients)

   ______________________________________________

   _______________________________________________

   ______________________________________________

   Oseltamivir (for patient who are tested positive for H1N1)

   Total number of days received:

   Dosage and Frequency:

   Treatment Group:

   ☐Treatment Arm: A (Hydroxychloroquine 400mg BD on day1 followed by 400 mg once daily for 10 days + Standard Treatment ST_s_)

   ☐Treatment Arm: B (Lopinavir(400mg) + Ritonavir(100mg) two tablets twice daily for 14 days+STs)

   ☐Treatment Arm: C (Lopinavir(400mg) + Ritonavir (100mg)+ Ribavirin)

   **CLINICAL FEATURES** (Unk = Unknown)

   | Cough and sputum production  Sore throat  Chest pain  Shortness of breath  Confusion | ☐Yes ☐No ☐Unk  ☐Yes ☐No ☐Unk  ☐Yes ☐No ☐Unk  ☐Yes ☐No ☐Unk  ☐Yes ☐No ☐Unk | Seizures  Vomiting  Nausea  Diarrhoea  Conjunctivitis  Myalgia  Other, specify_______ | ☐Yes ☐No ☐Unk  ☐Yes ☐No ☐Unk  ☐Yes ☐No ☐Unk  ☐Yes ☐No ☐Unk  ☐Yes ☐No ☐Unk  ☐Yes ☐No ☐Unk  ☐Yes ☐No ☐Unk |
   | --- | --- | --- | --- |

   **LABORATORY RESULTS ON FOLLOW UP** (*record units if different from those listed)

   | Parameter | Value | Not done | Parameter | Value | Not done |
   | --- | --- | --- | --- | --- | --- |
   | Haemoglobin (g/L) |  |  | Urine R/M (significant if any) |  |  |
   | WBC count (x109/L |  |  | Sodium (mEq/L |  |  |
   | Haematocrit (%) |  |  | Potassium (mEq/L) |  |  |
   | Platelets (x109/L) |  |  | Calcium(mg/dL) |  |  |
   | APTT/PT/INR |  |  | Lactate (mg/dL) |  |  |
   | Total bilirubin (mg/dL) |  |  | Procalcitonin (ng/mL) |  |  |
   | Direct bilirubin (mg/dL) |  |  | CRP (mg/L) |  |  |
   | Albumin (g/dl) |  |  | LDH (U/L) |  |  |
   | Globulin (g/dl) |  |  | Creatine kinase (U/L) |  |  |
   | SGOT/SGPT (U/L) |  |  | Troponin (ng/mL) |  |  |
   | Urea (mg/dL) |  |  | ESR (mm/hr) |  |  |
   | Creatinine (mg/dL) |  |  | D-dimer (mg/L) |  |  |
   | Others |  |  |  |  |  |

   **Influenza virus**: ☐Positive ☐Negative ☐Not done If positive, type ______________ **Coronavirus:** ☐Positive ☐Negative ☐Not done If positive: ☐MERS-CoV ☐SARS-CoV-2 ☐Other **Other respiratory pathogen**: ☐Positive ☐Negative ☐Not done If positive, specify:____________

   **Other pathogen of public health interest detected**: If yes, specify: __________________________

   **Radiological Investigations** (if performed):

   Chest X Ray/CT Performed (report):

   Ultrasonography:

   Others

   **SUPPORTIVE CARE** Is the patient CURRENTLY receiving any of the following?

   | ICU or High Dependency Unit admission? Yes No Unknown  Date of ICU/HDU admission [__][__]/[__][__]/[__][__][__][__] , ☐Unknown  ICU/HDU discharge date [__][__]/[__][__]/[__][__][__][__],☐Not discharged yet ☐Unknown  Oxygen therapy? ☐Yes ☐No ☐Unknown If yes, complete all below:  O2 flow volume: ☐1-5 L/min ☐6-10 L/min ☐11-15 L/min ☐>15 L/min ☐Unknown  Source of oxygen: ☐Piped ☐Cylinder ☐Concentrator ☐Unknown  Interface: ☐Nasal prongs ☐HF nasal cannula ☐Mask ☐Mask with reservoir ☐CPAP/NIV mask ☐Unknown  Non-invasive ventilation? (e.g. BIPAP, CPAP) ☐Yes ☐No ☐Unknown  Invasive ventilation (Any)? ☐Yes ☐No ☐Unknown  Inotropes/vasopressors? ☐Yes ☐No ☐Unknown  Prone position? ☐Yes ☐No ☐Unknown  Renal replacement therapy (RRT) or dialysis? ☐Yes ☐No ☐Unknown |
   | --- |

   **Previous Treatment Regime** (If any-to specify group with duaration):

   ☐Non-Severe Treatment arms (NS-group): Treatment Arm-A:___________________________

   ☐Non-Severe Treatment arms (NS-group): Treatment Arm-B:___________________________

   ☐Severe Treatment arms (S-group): Treatment Arm-A:___________________________

   ☐Severe Treatment arms (S-group): Treatment Arm-B:___________________________

   **Outcomes:**

   Clinical recovery ☐Yes ☐No

   Time to 2019-nCoV RT-PCR negative in upper respiratory tract specimen in days (if applicable):

   Time to defervescence in hours(if applicable)

   Serious adverse events (specify if any)

   **Final Clinician’s Impression:**

   **Adverse Event Log**

   | # | Date Reported | Adverse event description | Start Date | End Date | Outcome | Severity/  Grade | AE Treatment | Action Taken | PI Initials | Date of PI Initials |
   | --- | --- | --- | --- | --- | --- | --- | --- | --- | --- | --- |
   |  |  |  |  |  |  |  |  |  |  |  |
   |  |  |  |  |  |  |  |  |  |  |  |
   |  |  |  |  |  |  |  |  |  |  |  |
   |  |  |  |  |  |  |  |  |  |  |  |
   |  |  |  |  |  |  |  |  |  |  |  |
   |  |  |  |  |  |  |  |  |  |  |  |
   |  |  |  |  |  |  |  |  |  |  |  |

   | **Outcome** | **Severity/Grade** | **AE Treatment** | **Action Taken  with Study Intervention** |
   | --- | --- | --- | --- |
   | 0 – Fatal | 1 – Mild | 0 – None | 0 – None |
   | 1 – Not recovered/not resolved | 2 – Moderate | 1 – Medication(s) | 1 – Interrupted |
   | 2 – Recovered w/sequelae | 3 – Severe | 2 – Non-medication TX | 2 – Discontinued |
   | 3 – Recovered w/o sequelae | 4 – Life Threatening |  | 3 – Dose reduced |
   | 4 – Recovering/Resolving | 5 – Death (Fatal) |  | 4 – Dose increased |
   |  |  |  | 5 – Not Applicable |

   [↑](#endnote-ref-1)
